# Supplementary material for: Functional Genomics Analysis to Disentangle the Role of Genetic Variants in Major Depression
Source: Genes (Basel). 2022 Jul 15;13(7):1259. doi: 10.3390/genes13071259 (PMC9320424; doi:10.3390/genes13071259)
Supplement: Supplementary file 1 [file genes-13-01259-s001.zip › Perez-Granado-Supplementary_file_v2_after_proofreading.pdf]

Supplementary files for the article: Functional genomics analysis to disentangle the role of genetic variants in major depression

Supplementary Scheme S1

(this file)

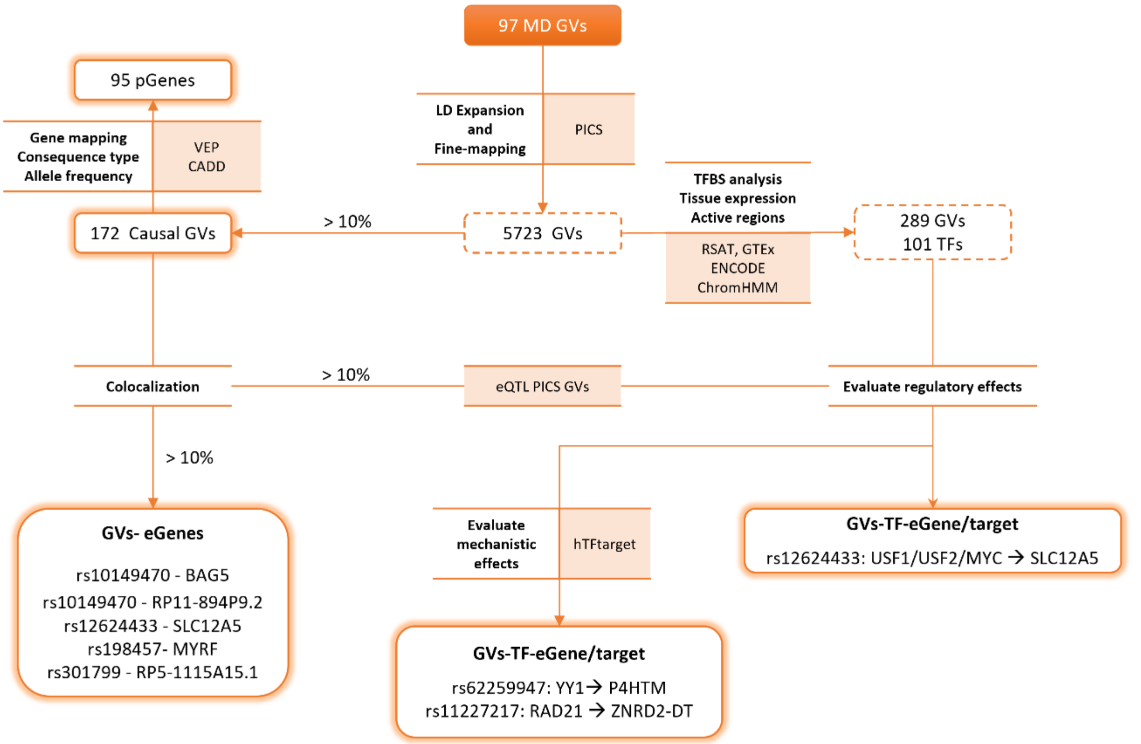

Supplementary Scheme S1. Study overview. Overview of the developed and implemented analysis pipeline including: fine-mapping, eQTL colocalization and TF binding analysis. eQTL: expression quantitative trait loci; TF: transcription factor; MD: major depression; GV: genetic variant; pGenes: proximal genes; VEP: Variant Effect Predictor; CADD: Combined Annotation Dependent Depletion; eGenes: genes regulated by eQTLs; PICS: Probabilistic Identification of Causal single nucleotide polymorphisms; RSAT: Regulatory Sequence Analysis Tools; GTEx: Genotype-Tissue Expression; ENCODE; Encyclopedia of DNA Elements.

Supplementary Figures S1-S3

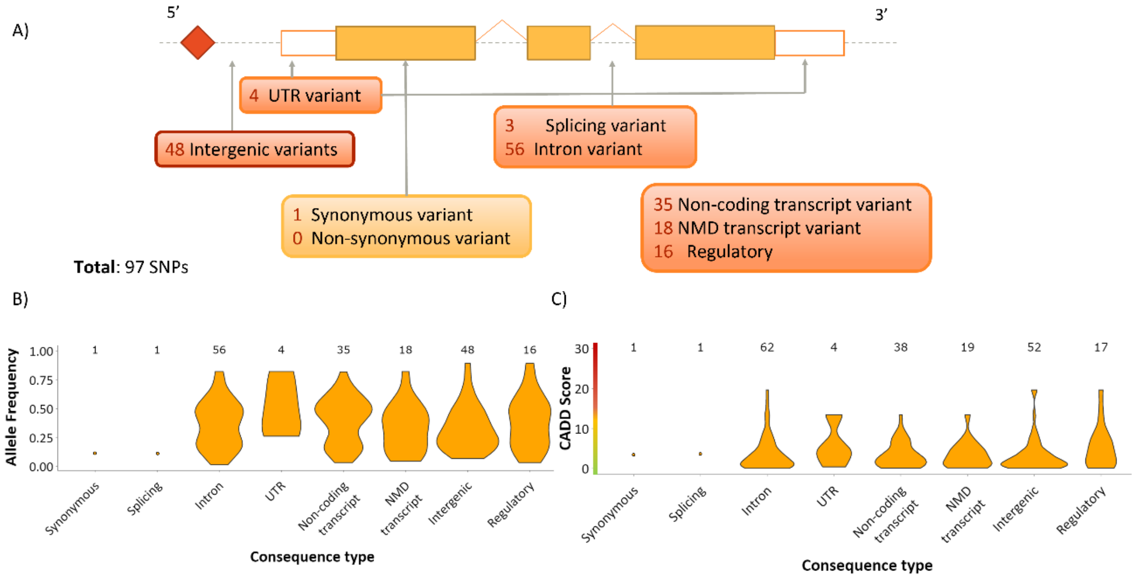

Supplementary Figure S1. MD lead GVs characterization. A) GVs distributed along the genome according to its consequence type predicted with VEP. B) Allele frequency density, according to GV's consequence type, also predicted with VEP. C) Pathogenicity score (predicted by CADD) density per consequence type. MD: major depression; GV: genetic variant; VEP: Variant Effect Predictos; CADD: Combined Annotation Dependent Depletion; UTR: untranslated region; NMD: Nonsense Mediated Decay

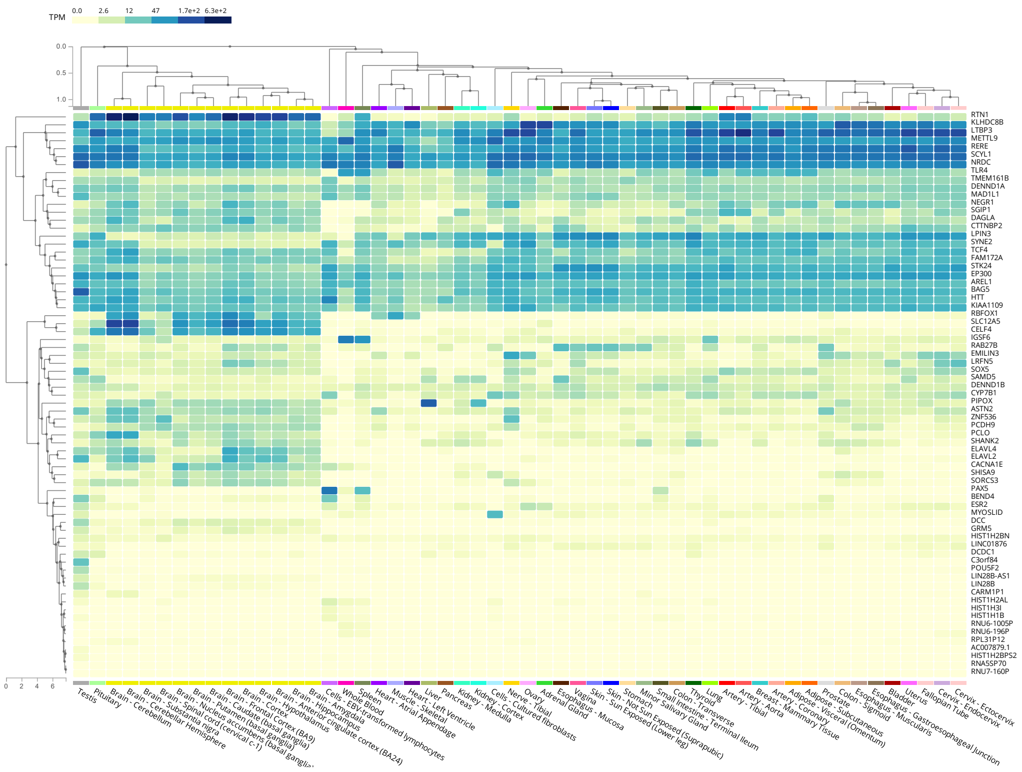

Supplementary Figure S2. Tissue expression of pGenes. Heatmap of pGenes tissue expression according to GTEx expression data (V8). pGenes: proximal genes; GTEx: Genotype-Tissue Expression.

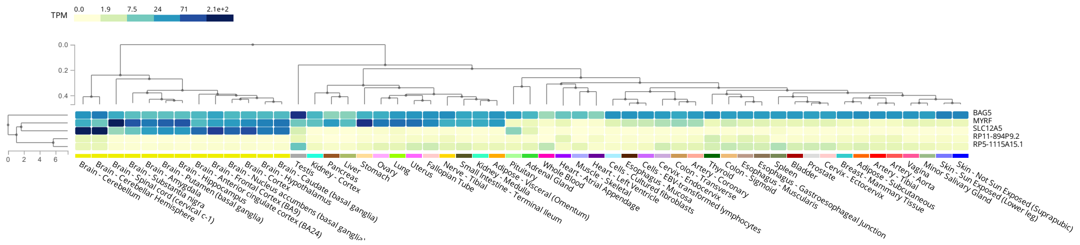

Supplementary Figure S3. Tissue expression of eGenes. Heatmap of eGenes tissue expression according to GTEx expression data (V8). eGenes: genes regulated by expression quantitative trait loci; GTEx: Genotype-Tissue Expression.

Supplementary Tables S1-S6

File: Perez-Granado-Supplementary_tables_1-6.xlsx

Supplementary Table S1. Summary of resources.

Supplementary Table S2. Causal GVs for MD.

Supplementary Table S3. pGenes functional and disease enrichment analysis.

Supplementary Table S4. Fine-mapped MD causal GVs disease enrichment analysis.

Supplementary Table S5. Colocalizing GWAS-eQTLs association to disease.

Supplementary Table S6. TFBS analysis.

Supplementary Table S7. GVs state annotation.
